# IRIS U kit usefulness in transanal total mesorectal excision for lower rectal cancer to avoid urethral injury

**DOI:** 10.1186/s12876-024-03279-8

**Published:** 2024-06-17

**Authors:** Masatsugu Ishii, Toshikatsu Nitta, Jun Kataoka, Yasuhiko Ueda, Ayumi Matsutani, Masataka Taki, Ryutaro Kubo, Masato Ota, Takashi Ishibashi

**Affiliations:** 1 Division of Surgery Gastroenterological Center, Medico Shunju Shiroyama Hospital, 2-8-1 Habikino, Habikino, Osaka 583-0872 Japan; 2 The Department of Colorectal Surgery, National Cancer Center Hospital East, Kashiwa, Japan

**Keywords:** IRIS U kit, Transanal, Rectal cancer, Urethral injury

## Abstract

Transanal total mesorectal excision (taTME) has improved the laparoscopic dissection for rectal cancer in the narrow pelvis. Although taTME has more clinical benefits than laparoscopic surgery, such as a better view of the distal rectum and direct determination of distal resection margin, an intraoperative urethral injury could occur in excision ta-TME. This study aimed to determine the feasibility and efficacy of the ta-TME with IRIS U kit surgery. This retrospective study enrolled 10 rectal cancer patients who underwent a taTME with an IRIS U kit. The study endpoints were the safety of access (intra- or postoperative morbidity). The detectability of the IRIS U kit catheter was investigated by using a laparoscope-ICG fluorescence camera system. Their mean age was 71.4±6.4 (58–78) years; 80 were men, and 2 were women. The mean operative time was 534.6 ± 94.5 min. The coloanal anastomosis was performed in 80%, and 20% underwent abdominal peritoneal resection. Two patients encountered postoperative complications graded as Clavien–Dindo grade 2. The transanal approach with IRIS U kit assistance is feasible, safe for patients with lower rectal cancer, and may prevent intraoperative urethral injury.

## Introduction

Transanal total mesorectal excision (taTME) is a breakthrough surgical procedure for treating lower rectal cancer. taTME is effective for dissection of the pelvic part in cases with narrow pelvis cavity, obesity, and bulky tumors. However, it needs adequate dissection through the external and internal sphincter muscle layers. Furthermore, there is no clear landmark, and the risk of urethral injury exists during the dissection anterior rectal wall. Thus, using the infrared illumination system urethral kit (IRIS U kit) during the dissection anterior rectal wall in ta-TME is important because it provides a colored borderline between the urethral tract and anterior rectal wall. Therefore, this study aimed to explore the effectiveness of ta-TME with the IRIS U kit to avoid urethral injury.

## Materials and methods

This single-centered retrospective study enrolled 10 patients with biopsy-proven rectal cancer from rectal tumors who underwent minimum laparoscopic-assisted rectal surgery at the Shiroyama Hospital in Japan from January 2019 to September 2021. The study was conducted according to the relevant guideline and regulations. We have the approval of the ethics and committee (committee number is 2018-004), and the consents from patients and ethics. The institutional review board of the Shiroyama Hospital of Osaka approved the IRIS U-kit trans anal resection use for rectal cancer in humans. Written informed consent was obtained from all patients. Oncological principles of surgical resection for rectal cancer were followed. Pre-operative diagnosis and staging were carried out in all cases with colonoscopy and biopsy, enema contrast examination, abdominal and pelvic computed tomography, and magnetic resonance imaging. Neoadjuvant chemotherapy treatment was administered to four patients (Table 1). Oxaliplatin once every 2 weeks and chemotherapy, which included continuous 5-fluorouracil (5-FU) infusion for 2 days, were administered and were well-tolerated by all patients. We waited 4 and 6 weeks to complete neoadjuvant therapy before performing the surgery. Patients’ tumor characteristics and subjective demographics are provided in (Table 1).

**Table 1:** Characteristics of preoperative patients

Characteristic	Value
Age at surgery, years, mean ± SE (range)	73(58–78)
Sex	
Male	9 (82%)
Female	2 (18%)
BMI kg/m^2^, mean ± SE (range)	22 (15–34)
ASA classification	
II	2 (2–3)
Location	Rb
The range of distance from anal verge, cm	3.3 (2–4.5)
Neoadjuvant Chemotherapy, case	5 (45%)

### Preoperative preparation

Prophylactic antibiotics (cefmetazole 2 g) were administered intravenously, and a thoracic epidural catheter was inserted for pain control. The patients were in lithotomy position with bilateral arms fixed to the sides.

### Surgical procedure

**Fig. 1:**
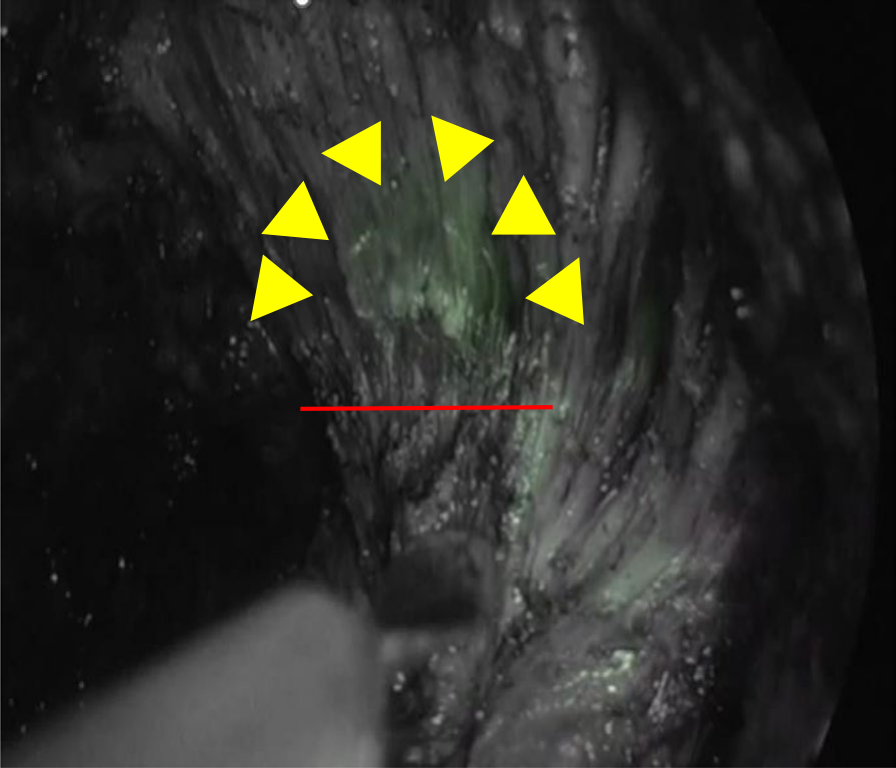
IRIS U kit catheter system shows that an important demarcation line (red line) between the anterior rectal wall and the urethral duct (yellow arrow) could be visualized during transanal dissection in ta-TME ICG mode

First, the abdomen was insufflated to a pressure of 10 mm Hg via a balloon port inserted through the umbilicus, and a 10 mm port was inserted through the right lower incision for a 30° angle laparoscope (10 mm balloon key port). A total of three ports were inserted in the left upper and lower, and right upper quadrants. Then, the small intestine was moved from the upper space of the pelvis, and no liver metastasis, peritoneal dissemination, and other coarse lesion were observed in the abdominal space. Combined transrectal and laparoscopic dissection for all cases were performed using a multiport rectal device (Gel POINT Path or Mini, Transanal; Applied Medical, USA) that was inserted and sealed; CO2 was insufflated to a pressure of 10 mm Hg. Next, a 30° angle video laparoscope (Stryker) was introduced through the single port device for direct viewing. A purse-string suture was then placed through the rectal mucosa to tightly occlude the rectum with a 2–3 cm margin distal from the tumor using the Lone Star Retractor System. Distal to the purse string, a full-thickness rectal transection was initiated circumferentially. Once within the presacral plane, the mesorectum was mobilized, and the posterior dissection proceeded cephalad in the avascular presacral plane in accordance with total mesorectal excision (TME) principles. Subsequently, this plane of dissection was extended medially and laterally, with careful maneuvering of the vagina or prostate from the anterior rectal wall to achieve circumferential rectal mobilization. The peritoneal rectal attachments were then divided transanally, and the peritoneal cavity was entered (Fig. 1). Laparoscopic graspers were used to retract and aid the dissection of the rectosigmoid and expose the vascular pedicle. The inferior mesenteric vessels were then transected at their base with vascular clips.

### Anastomoses and diverting or permanent stoma

Furthermore, after confirming that sufficient length of the colon had been freed, the transanal single port was removed, the Lone Star Retractor was then positioned, and the rectosigmoid was carefully exteriorized transanally. In all cases, the specimen was removed transanally. Proximal colonic resection was performed extracorporeally. In eight patients, hand-sewn coloanal anastomoses were performed between the proximal sigmoid colon and distal anorectal cuff. The anastomoses were tested using the ICG blood flowing test. In seven patients, a temporary loop ileostomy was created and matured in a standard fashion. One patient had no stoma, because we performed the pull-through method. In two patients, permanent colostomy was created for abdominal peritoneal resection, and the right lower quadrant mini port site was used. For all patients, a suction drain was placed in the deep pelvis and exteriorized through the left lower quadrant mini port site.

### Endpoint parameters

The Clavien-Dindo were used to classify the endpoint parameters to meet the oncological resection criteria (TME, distal and circumferential margins) and access safety (as measured by complications).

## Results

From January 2020 to October 2021, a total of 10 consecutive patients underwent ta-TME, and 10 patients underwent surgery in Shiroyama Hospital, Habikino City, in Japan. The mean age of patients was 73 (58–78) years, and 80% (8 patients) were men. The follow-up data for 24 months was complete for all patients. Neoadjuvant chemotherapy was administered in 4 patients (40%), and 6 (60%) underwent primary anastomosis during surgery. Two (20%) patients encountered postoperative complications with Clavien–Dindo grade II. Patient characteristics and short-term clinical outcomes are summarized in Tables 1 and 2.

**Table 2:** Pathological characteristics of patients during the operative stage

Characteristics	Value
Surgical procedure	
taTME/ISR	5
taTME/ISR/Pull-through	1
taTME/APR/LLND	2
taTME/ISR/LLND	3
Operative time, min, mean ± SE (range)	525(431–766)
Bleeding, mL, mean ± SE (range)	180 (10–330)
Anastomosis	
Hand-sewn coloanal	9
Protective ileostomy	8
ASA classification	
II	2 (2–3)
Location	
Rb	9
Rb/RS	1
RbP	1
Pathological stages	
I	3
II a	3
IIc	1
IIIa	0
IIIb	2
IIIc	2
The number of lymph nodes harvested mean ± SE (range)	25 (1-57)
Dsital margin±SE (range), mm	30 (10-75)
Proximal margin mean±SE (range), mm	81 (35-230)
Postoperative complications	
Outlet obstruction	1
Paralytic ileus	1

*ISR* Intersphincteric resection, *APR* Abdominal peritoneal resection, *LLND* Lateral lymph node dissection, *ASA* American Society of Anesthesiologists, *TaTME* Transanal total mesorectal excision, *Rb* lower rectum, *Rb/RS* lower rectum/upper rectum, *RbP* lower rectum, anal canal

A laparoscopic approach was performed for all procedures. The mesorectal plane was transanally dissected entirely up to the level of peritoneal reflection in the superior pelvis. Complete mobilization of the splenic flexure was required in one case to assist laparoscopy. The main operative and pathological characteristics of patients are listed in Table 2. Pathologic analysis confirmed that distal margins were free of tumor. Using the Clavien–Dindo classification, 8 out of 10 (80 %) patients had no complications, and 2 patients (20 %) had at least one. There was no postoperative mortality rate (grade V), while two patients had grades I (10 %) and II complications and urinary infection, and 1 (10 %) had grade II complications with postoperative ileus, while others had severe dehydration due to increased ileostomy output.

## Discussion

The conventional laparoscopic resection procedures for lower rectal cancer are difficult to perform due to an adequate operative field in patients with obesity, narrow pelvis, or bulky tumor. Furthermore, it is challenging to determine the rectal wall with the urethral tract. taTME is a new surgical procedure for lower rectal cancer [1]. This is an efficient method for dissecting intrapelvic resection with obesity, narrow pelvic space, and bulky tumor than the conventional laparoscopic transabdominal approach [2, 9, 15–17]. The approach is effective because the laparoscopic view is straight during the dissection of the anterior rectal wall [9–14]. However, there is no clear landmark during the dissection of the anterior rectal wall and urethral tract [3]. Selecting the surgical approach depends on the patient body habitus (obesity or narrow pelvis), tumor status (location and extent), and surgeon’s preference and experience. This technique was developed because of the limitations in the deep pelvis of laparoscopic surgery, especially in western countries. Our first ta-TME with the IRIS U kit for rectal cancer was performed on a 58-year-old man with rectal cancer 4 cm from the anal verge. ta-TME that used IRIS U kit might avoid urethral injury to perform rectal resections. In this study, we have developed the technique of transanal surgery assisted by a two-team laparoscopy [4]. In ta-TME, the urethral injury was reported to occur in 2–6% of cases [5–7]. Strategies involving accurate anatomical navigation have been investigated to avoid urethral injury. For example, Atallah et al. [8] suggested real-time and convenient navigation during operation. Moreover, A cadaveric study described that retrograde direct intraurethral ICG injection is effective for detection of the urethra [18].

**Fig. 2:**
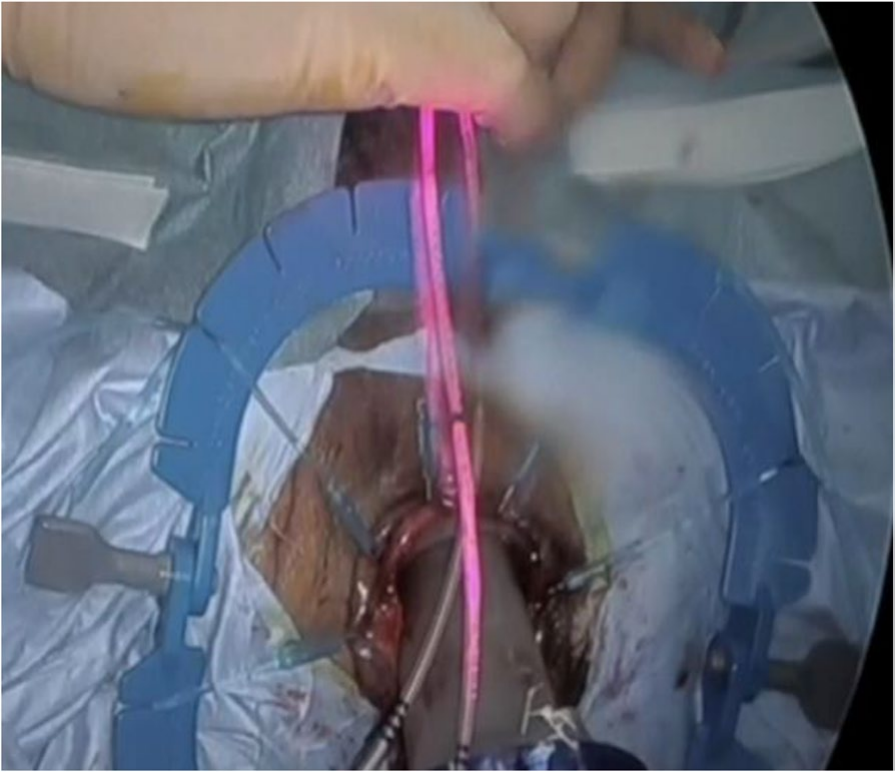
We can distinguish the prostatic segment of the urethra in real-time by using the infrared illumination system urethral kit (IRIS U kit)

Our experiment proved that the IRIS U kit catheter system could be used for real-time and clear navigation that changes color to distinguish segments. During the dissection of the anterior area among the rectal wall and recto-urethral muscle, we placed the IRIS U kit catheter wire through the urethra. The endoscopic near-infrared (NIR) visualization light increases visibility when using the laparoscopic system (1588 or 1688AIM™; Stryker) during the laparoscopic and taTME stages of the procedure. We can distinguish the prostatic segment of the urethra in real-time by using the infrared illumination system urethral kit (IRIS U kit) (Fig. 2) under endoscopic NIR visualization. The IRIS U kit catheter system is a simple and non-invasive technique and may become a useful and safe option. Our study shows that an important demarcation line between the anterior rectal wall and the urethral duct could be visualized during transanal dissection. In our 10 patients, we found that this approach with the luminal item was feasible and convenient to perform complete rectal dissection with moderate ta-TME and harvest a sufficient number of dissected lymph nodes. In our hospital, laparoscopic ta-TME has been performed without critical complications for the treatment of rectal cancer, even with lateral lymph node metastasis or invasion to the adjacent organ. Pathological results revealed that the distal margin was negative in all patients; however, circumferential margins were positive in three. The stage of the 3 patients’ pathological depth was T4. Approximately 8 out of 10 patients had no complications. The short-term oncologic outcomes of this series confirmed the oncologic and technical safety of the transanal approach for rectal cancer. We believe this approach has an advantage when performing the rectal anterior wall dissection, especially in men with obesity in the pelvic space. Our experiments proved that real-time navigation using the IRIS U kit catheter system is possible during rectal anterior dissection in ta-TME. This study has limitations. First, this had a small sample size. Second, this was a single-center study without a control group. Therefore, the feasibility and usefulness of the IRIS U kit catheter system in ta-TME should be further assessed in a clinical trial.

In conclusion, the IRIS U kit catheter system is safe and feasible in avoiding intraoperative urethral injury during the rectal wall and urethral tract dissection in ta-TME. ta-TME may become a standard surgical treatment procedure for rectal cancer because the urethral tract can be easily detected through the intraoperative view.

## Data Availability

No datasets were generated or analysed during the current study.

## Abbreviations

taTME: Transanal total mesorectal excision
IRIS U kit: Infrared illumination system urethral kit
ICG: Indocyanine green

## References

[1] deLacy AM, Rattner DW, Adelsdorfer C, Tasende MM, Fernandes M, Delgado S, Sylla P, Martines-Palli G (2013). Transanal natural orifice transluminal endoscopic surgery (NOTES) rectal resection: “Down-to-up” total mesorectal excision (TME)–-short-term outcomes in the first 20 cases. Surg Endosc.

[2] Araujo SE, Crawshaw B, Mendes CR, Delaney CP (2014). Transanal total mesorectal excision: a systematic review of the experimental and clinical evidence. Tech Coloproctol.

[3] Nakajima Y, Muro S, Nasu H, Harada M, Yamaguchi K, Akita K (2017). Morphology of the region anterior to the ana canal in males: visualization of the anterior bundle of the longitudinal muscle by transanal ultrasonography. Surg Radiol Anat.

[4] Nitta Toshikatsu, Tanaka Keitaro, Kataoka Jun, Ohta Masato, Ishii Masatsugu, Ishibashi Takashi, Okuda Junji (2019). Novel technique with the IRIS U kit to prevent urethral injury in patients undergoing transanal total mesorectal excision. Ann Med Sur (Lond).

[5] Atallah S (2015). Transanal total mesorectal excision: full steam ahead. Tech Coloproctol.

[6] Kang L, Chen WH, Luo SL, Luo YX, Liu ZH, Huang MJ, Wang JP (2016). Transanal total mesorectal excision for rectal cancer : a preliminary report. Surg Endosc.

[7] Rouanet P, Mourregot A, Azar CC, Carrere S, Gutowski M, Quenet F, Saint-Aubert B, Colombo PE (2013). Transanal endoscopic proctectomy: an innovative procedure for difficultresection of rectal tumors in men with narrow pelvis. Dis Colon Rectum.

[8] Attallah S, Martin-Perez B, Larach S (2015). Image-guided real-time navigaton for transanal total mesorectal excision: a pilot study. Tech Coloproctol.

[9] Sylla P, Rattner DW, Delgado S, Lacy AM (2010). NOTES transanal rectal cancer resection using transanal endoscopic microsurgery and laparoscopic assistance. Surg Endosc.

[10] Lacy AM, Saavedra-Perez D, Bravo R, Adelsdorfer C, Aceituno M, Balust J (2012). Minilaparoscopy-assisted natural orifice total colectomy: technical report of a minilaparoscopy-assisted transrectal resection. Surg Endosc.

[11] Lacy AM, Adelsdorfer C, Delgado S, Sylla P, Rattner DW (2013). Minilaparoscopy-assisted transrectal low anterior resection (LAR): a preliminary study. Surg Endosc.

[12] Pearl JP, Marks JM, Ponsky JL (2008). Hybrid surgery: combined laparoscopy and natural orifice surgery. Gastrointest Endosc Clin N Am.

[13] Horgan S, Cullen JP, Talamini MA, Mintz Y, Ferreres A, Jacobsen GR, Sandler B, Bosia J, Savides T, Easter DW, Savu MK, Ramamoorthy SL, Whitcomb E, Agarwal S, Lukacz E, Dominguez G, Ferraina P (2009). Natural orifice surgery: initial clinical experience. Surg Endosc.

[14] Guillou PJ, Quirke P, Thorpe H, Walker J, Jayne DG, Smith AM, Heath RM, Brown JM; MRC CLASICC Trial Group (2005). Short-term endpoints of conventional versus laparoscopic assisted surgery in patients with colorectal cancer (MRC CLASICC trial): multicentre randomised controlled trial. Lancet.

[15] Chen YT, Kiu KT, Yen MH, Chang TC (2019). Comparison of the short-term outcomes in lower rectal cancer using three different surgical techniques: transanal Total Mesorectal Excision (TME), laparoscopic TME, and open TME. Asian J Surg..

[16] Jang HB, Kang SB, Lee H, Choi BJ, Lee SC (2022). Anastomotic leakage and chroic presacral sinus after transanal total mesorectal excision (taTME) for rectal cancer: a comparative study to laparoscopic TME. Asian J Surg..

[17] Hu JM, Chu CH, Jiang JK, Lai YL, Huang IP, Cheng AY, Yang SH, Chen CC (2020). Robotic transanal total mesorectal excision assisted by laparoscopic transabdominal approach: A preliminary twenty-case series report. Asian J Surg..

[18] Barnes TG, Penna M, Hompes R, Cunningham C (2017). Fluorescence to highlight the urethra: a human cadaveric study. Tech Coloproctol.

